# Mobile bearing total knee arthroplasty does not lead to better joint awareness compared to fixed bearing design: A systematic review and meta‐analysis

**DOI:** 10.1002/jeo2.70110

**Published:** 2024-12-15

**Authors:** Mohammad Poursalehian, Yeganeh Pakbaz, Seyed Mohammad Javad Mortazavi

**Affiliations:** ^1^ Joint Reconstruction Research Center Tehran University of Medical Sciences Tehran Iran

**Keywords:** fixed‐bearing total knee arthroplasty, Forgotten Joint Score‐12, joint awareness, mobile‐bearing total knee arthroplasty

## Abstract

**Purpose:**

Mobile‐bearing total knee arthroplasty (MB‐TKA) and fixed‐bearing (FB) TKA are both widely used, with MB‐TKA theoretically offering better functional outcomes due to its natural kinematics. This systematic review and meta‐analysis aimed to compare joint awareness between MB‐TKA and FB‐TKA, as measured by Forgotten Joint Score‐12 (FJS‐12), to provide insights into patient‐perceived outcomes.

**Methods:**

A comprehensive literature search was conducted across major databases following PRISMA guidelines, without date or language restrictions. Studies focusing on TKA with MB or FB as the intervention and control groups, respectively, and reporting on FJS‐12 were included. The selection process involved two independent reviewers. Data extraction was carried out using a structured checklist and assessed for quality using the Newcastle–Ottawa Scale (NOS). The meta‐analysis employed Hedge's *g* method to compare FJS‐12 and assessed publication bias using Egger's test and funnel plot analyses.

**Results:**

Six studies, including two randomized clinical trials and four cohort studies with 731 participants and mean follow‐up of 5.4 years, met the inclusion criteria. The meta‐analysis revealed no significant difference in FJS‐12 between MB and FB TKA (pooled difference = 0.132, 95% confidence interval: −0.103 to 0.367, *p* = 0.271), with moderate heterogeneity observed (*I* ^2^ = 53.5%). Publication bias assessment indicated no significant bias. Meta‐regression did not identify factors contributing to heterogeneity.

**Conclusion:**

MB‐TKA does not provide superior patient‐perceived outcomes in terms of joint awareness compared to FB‐TKA. This suggests that the clinical advantage of MB‐TKA in terms of joint awareness is likely negligible. Therefore, the choice between MB and FB TKA should be based on other considerations, such as surgeon preference, implant cost and individual patient needs.

**Level of Evidence:**

Level III.

## Graphical abstract

Joint awareness is the same in mobile and fixed bearing TKA

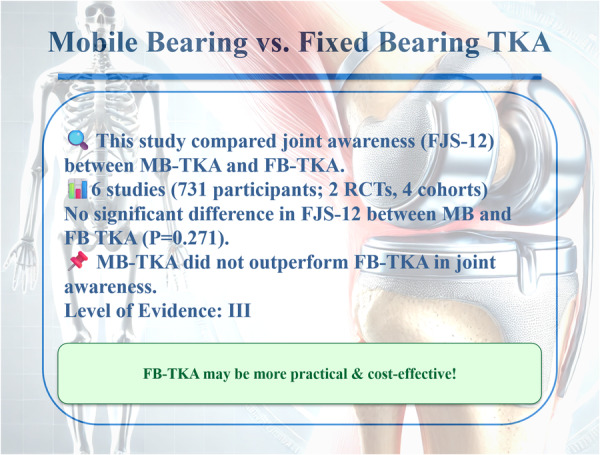

**Abbreviations**
BMI: body mass index
FB: fixed‐bearing
FJS‐12: Forgotten Joint Score‐12
MB‐TKA: mobile‐bearing total knee arthroplasty
NOS: Newcastle–Ottawa Scale
PRISMA: Preferred Reporting Items for Systematic Reviews and Meta‐Analyses
PROM: patient‐reported outcome measure
PROSPERO: International Prospective Register of Systematic Reviews
RCT: randomized clinical trial
TKA: total knee arthroplasty

## INTRODUCTION

As medical technology advances, various surgical techniques and prosthetic designs have been developed to enhance patient outcomes and satisfaction [15]. Among these, mobile‐bearing total knee arthroplasty (MB‐TKA) and fixed‐bearing (FB) TKA are prominent options, each with distinct mechanical and design features [6]. MB‐TKA is designed to closely mimic the natural kinematics of the knee, potentially offering better functional outcomes and inducing the same level of natural joint feeling [6]. The choice between MB‐TKA and FB‐TKA remains a topic of debate among orthopaedic professionals [29].

Previous meta‐analyses comparing MB and FB TKA generally found no significant differences between the two in terms of patient‐reported outcome measures (PROMs) such as the Knee Society Score (KSS), Western Ontario and McMaster Universities Arthritis Index (WOMAC) and range of motion, as well as survival rates [4, 7, 9, 10, 13, 26, 27, 28]. However, what sets the Forgotten Joint Score‐12 (FJS‐12) apart from other PROMs is its specificity in measuring joint awareness during daily activities, which has become an increasingly important factor in evaluating patient satisfaction with TKA [16]. Unlike conventional PROMs that assess pain, function and stability, the FJS‐12 focuses on the extent to which patients ‘forget’ their artificial joint throughout the day, offering a unique insight into the subjective experience of living with a joint replacement [22]. Higher FJS‐12 indicates lower joint awareness, which is associated with better patient outcomes in terms of perceived natural function. The FJS‐12 is particularly valuable for detecting subtle differences in patient‐perceived outcomes that may not be captured by traditional PROMs, largely due to its lower ceiling effects [12]. This allows it to more accurately reflect the patient experiences, especially in cases where other PROMs might indicate a plateau in perceived outcomes.

To the best of our knowledge, no previous meta‐analysis has used joint awareness as the primary outcome, leaving a gap in the literature on patient‐perceived outcomes following TKA. In this review, the importance of the FJS‐12 as a distinct outcome measure is emphasized, and it is hypothesized that the theoretical kinematic advantages of MB‐TKA may translate into superior joint awareness.

## METHODS

### Protocol and registration

This study is implemented according to the PRISMA (Preferred Reporting Items for Systematic Reviews and Meta‐Analyses) statement.

The FJS‐12 is a validated PROM specifically designed to assess a patient's awareness of their artificial joint during daily activities. Unlike traditional PROMs, the FJS focuses on the degree to which patients forget the presence of their joint replacement, which is considered a sign of successful joint function. This score consists of 12 items that assess joint awareness during various physical and social activities. The items are scored on a 5‐point Likert scale, with responses ranging from ‘never’ to ‘always’ (never = 0, almost never = 1, seldom = 2, sometimes = 3 and often = 4). The total score is then transformed into a 0–100 scale, where a higher score indicates a lower degree of joint awareness and thus a better outcome. This makes the FJS a unique tool for evaluating the long‐term functional success of joint replacement, as it captures patients' ability to ‘forget’ their joints in everyday life.

### Search strategy

On 1 February 2024, an extensive and systematic literature search was executed across four major databases: PubMed, Embase, Scopus and Web of Science. Our comprehensive search strategy incorporated a combination of key terms and Boolean operators, formulated as ‘Bearing’ AND (‘Total Knee Arthroplasty’ OR TKA) AND (‘Forgotten Joint Score’ OR FJS). No restrictions were imposed on the publication date or language of the articles, ensuring a wide‐ranging and inclusive search.

### Eligibility criteria

This systematic review will include studies that specifically focus on patients undergoing TKA. The population (P) of interest comprises individuals who have undergone TKA, without restrictions on demographics such as age, gender or underlying conditions. The intervention (I) considered for this review is the use of MB in TKA. Comparatively, the control (C) group will consist of patients who have undergone TKA with an FB. The primary outcome (O) of interest is the FJS. For study design (S), the review will include randomized clinical trials (RCTs) and Cohort studies. Studies will be excluded if they do not meet these criteria, if they do not provide clear data on the FJS, or if they focus on interventions other than MB or FB in the context of TKA.

### Study selection

The entire selection process was facilitated by Endnote X8 (Clarivate Analytics). The initial phase involved a thorough examination of titles and abstracts by two independent reviewers (MP and YP), ensuring an unbiased review. The subsequent phase entailed a detailed analysis of the full texts, focusing on identifying the most relevant articles that align with our research criteria. During the selection process, any discrepancies between reviewers were resolved through constructive discussions, and if necessary, the final decision was made through the consensus of the corresponding author (SMJM).

### Data extraction

A meticulously designed checklist was employed for data extraction to uniformly gather key study details. This checklist included variables such as the author's name, publication year, level of evidence, country of origin, type of alignment strategy, sample size, percentage of female participants, mean age, mean body mass index (BMI), duration of follow‐up, FJS‐12 and type of prosthesis used. Two independent researchers executed this data extraction process in Microsoft Excel (YP and MP). Any discrepancies observed were judiciously reviewed and reconciled by a third investigator (SMJM) (Table 1).

**Table 1:** Study characteristics.

Author, Year	Level of evidence	Country	Sample size	Lost patients to FU	Female percentage (%)	Mean age (years)	Mean BMI	Mean FU duration (years)	Alignment strategy	Any significant differences	Other outcomes	Undergone FB‐TKA			Undergone MB‐TKA		
												Sample size	Prosthesis	Mean FJS	Sample size	Prosthesis	Mean FJS
Thienpont, 2016 [23]	III	Belgium	100	NA	71	68.7	29.7	1.5	Mechanical	NA	KSS	50	Vanguard PS	71	50	Vanguard ROCC	57
Thomsen, 2016 [24]	III	Denmark	189	NA	60	63.9	NA	1.7	NA	NA	OKS	121	AGC	52	68	Vanguard ROCC	57
Bakircioglu, 2023 [1]	III	Turkey	140	NA	10	63.7	28.5	15.3	Mechanical	ROM	KSS, ROM, WOMAC	70	NexGen LPS	73	70	NexGen LPS	66
Kalaai, 2023 [8]	II	Netherlands	38	12	39	63.0	29.5	5	NA	NA	KSS, OKS, WOMAC, LEAS	18	Vanguard PS	63	20	Vanguard DDRP	73
Sohn, 2023 [19]	II	Korea	98	2	96	69.5	26	3	NA	NA	KSS, ROM, WOMAC, HKA	49	ACS PS	67	49	ACS PS	63
Ueyama, 2023 [25]	III	Japan	190	NA	88	76.0	26.1	5.2	Mechanical	FU	KSS, ROM	95	Vanguard PS	57	95	Vanguard RP	55

Abbreviations: AGC, anatomic graduated components; BMI, body mass index; DDRP, deep dish rotating platform; FB, fixed‐bearing; HKA, hip–knee–ankle; KSS, Knee Society Score; FU, follow‐up; LEAS, Lower Activity Extremity Score; LPA, legacy posterior stabilized; MB, mobile‐bearing; NA, not available; OKS, Oxford Knee Score; PS, posterior stabilizer; ROCC, ROtative Concave Convex; ROM, range of motion; TKA, total knee arthroplasty; WOMAC, The Western Ontario and McMaster Universities Arthritis Index.

### Quality assessment

**Figure 1:**
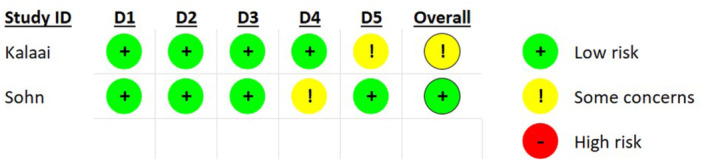
RoB2 quality assessment of RCTs. RCT, randomized clinical trial; RoB2, risk of bias tool version 2.

The Newcastle‐Ottawa Scale (NOS), a widely recognized tool for evaluating the methodological quality of non‐randomized studies in systematic reviews, was applied to assess the quality of the included studies (Table 2) [20]. For RCTs, the risk of bias tool version 2 (RoB2) was implemented to assess quality and bias (Figure 1) [21].

**Table 2:** Quality assessment of included studies.

Study	Selection	Comparability	Outcome	Overall quality
Thienpont, 2016	✹✹✹✹	✹✹	✹✹	Good
Thomsen, 2016	✹✹✹✹	✹✹	✹✹	Good
Bakircioglu, 2023	✹✹✹✹	✹✹	✹✹	Good
Ueyama, 2023	✹✹✹✹	✹✹	✹✹	Good

### Data synthesis

A meta‐analytic approach was adopted to quantitatively analyze and interpret the findings from the extracted data. The *I*² test was used to assess heterogeneity, and random‐effects models were employed to account for possible heterogeneity.

The statistical analysis was conducted using the Hedge's *g* method. This approach was selected due to its effectiveness in estimating the standardized mean difference, allowing for a more precise comparison of the FJS across different studies. This method is particularly beneficial when dealing with studies of varying sizes, as it adjusts for small sample bias.

To assess the presence of publication bias, both Egger's test and funnel plot analyses were utilized. Egger's test provided a statistical measure for detecting bias, while funnel plots offered a visual representation of any asymmetry in the meta‐analysis, potentially indicating bias. In cases where significant heterogeneity or potential sources of bias were detected, meta‐regression analyses were conducted. This advanced statistical method allowed for exploration and identification of potential factors contributing to the observed variability or bias in the study outcomes.

## RESULTS

### Study characteristics

**Figure 2:**
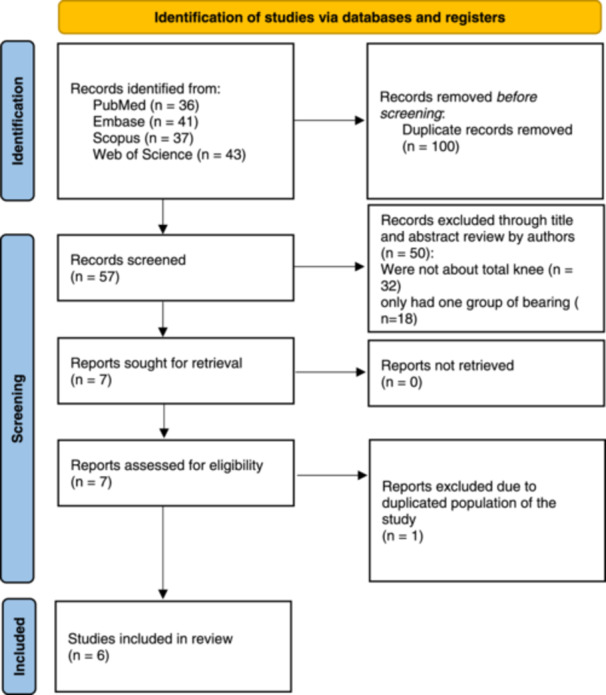
PRISMA flow chart illustrating the study selection process.

Our meticulous screening process of 157 articles culminated in the inclusion of six studies in this systematic review, as depicted in the PRISMA flow chart (Figure 2). Notably, during the data extraction phase, it was identified that one study was an extended follow‐up of another [8]. Consequently, the earlier study was excluded to avoid redundancy [18].

Of the final selection, two studies were RCTs [8, 19], and the remaining four were cohort studies [1, 23, 24, 25]. Collectively, these studies encompassed 731 participants, characterized by a mean age of 68.2 years and a mean follow‐up duration of 5.4 years. The participant pool was predominantly female, with 474 (62.8%) female participants. Geographically, these studies spanned various countries, including Belgium, Denmark, the Netherlands, Japan, Korea and Turkey. In terms of alignment principles, three studies (50%) utilized mechanical alignment, while the alignment strategy in the remaining three studies (50%) was unspecified. A detailed breakdown of these studies can be found in Table 1.

### Quality assessment

The quality assessment of the included studies, as shown in Table 2 and Figure 1, revealed that all studies were of high quality, indicating a robust and reliable body of evidence.

### Meta‐analysis

**Figure 3:**
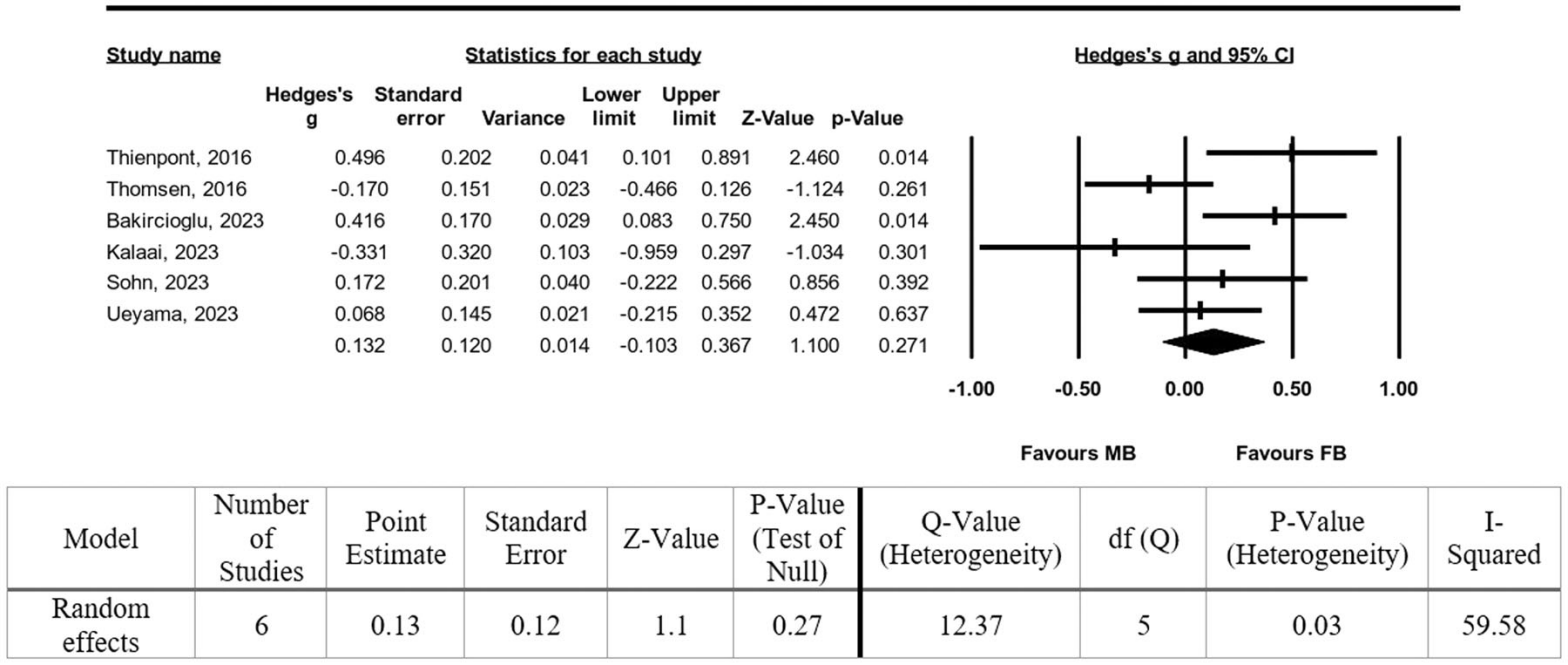
Forest plot of the pooled difference in FJS‐12. FJS‐12, Forgotten Joint Score‐12.

The pooled difference in the FJS‐12 across the included studies was 0.132 (95% confidence interval: −0.103 to 0.367) using Hedge's *g*. This difference, favouring FB, was not statistically significant (*p* = 0.271). The heterogeneity observed in these studies was moderate (*I* ^2^ = 53.5%). A detailed forest plot of this analysis is presented in Figure 3.

### Publication bias

**Figure 4:**
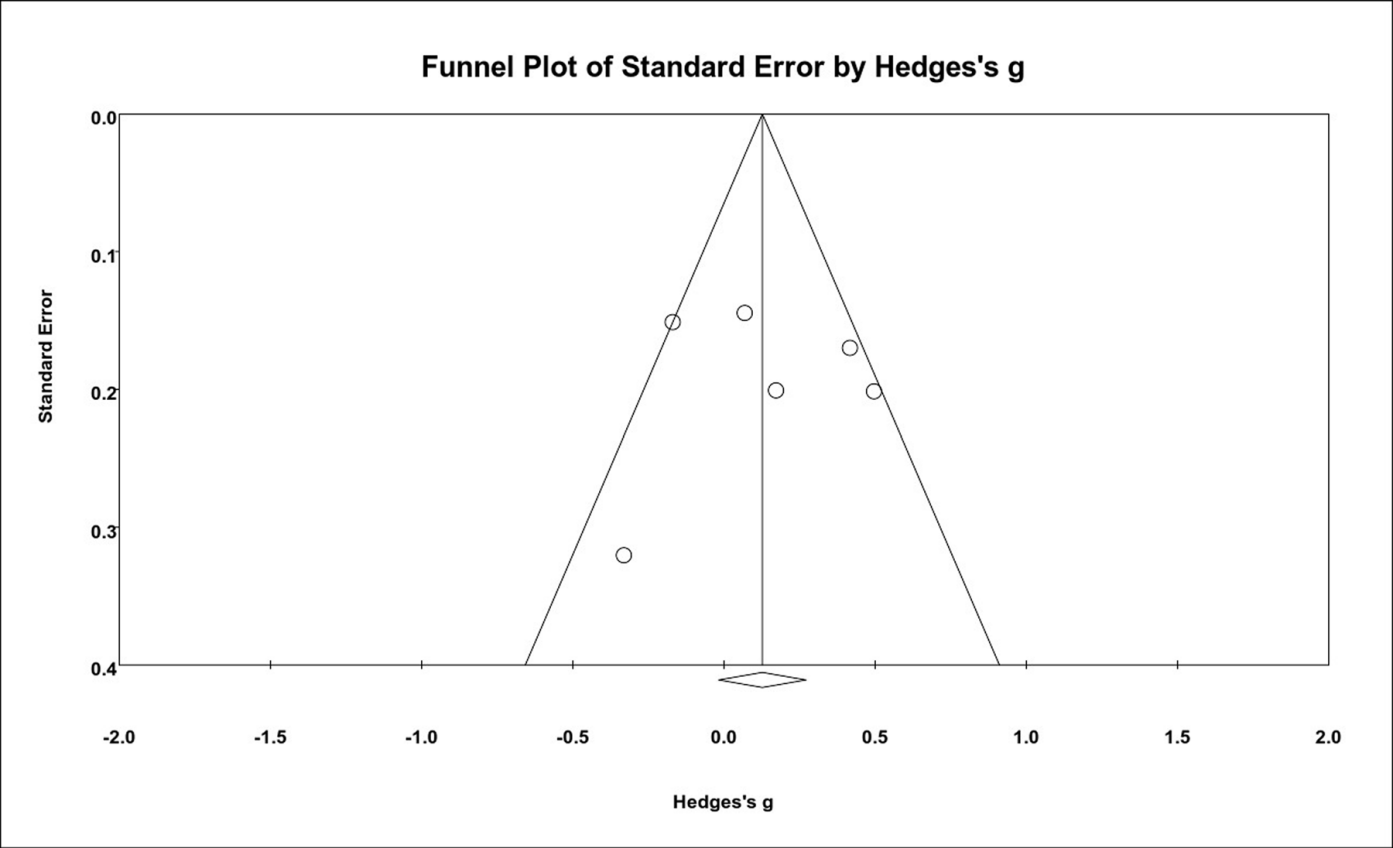
Funnel plot for assessment of publication bias.

The assessment of publication bias using Egger's test yielded a non‐significant result (*p* = 0.97), suggesting no substantial bias in the published literature. The funnel plot visualizing this analysis can be found in Figure 4.

### Meta‐regression

A meta‐regression analysis was conducted to explore the sources of the observed moderate heterogeneity. This analysis included variables such as mean age, female ratio, quality of studies, BMI and level of evidence. However, none of these factors significantly accounted for the heterogeneity observed among the included studies.

## DISCUSSION

The primary objective of this systematic review and meta‐analysis was to compare patient joint awareness, as measured by the FJS‐12, between MB‐TKA and FB‐TKA. Our findings revealed no statistically significant difference in joint awareness between the MB and FB designs.

The concept underlying MB‐TKA focuses on mimicking the knee's natural kinematics, theoretically leading to a joint that feels more natural and less noticeable to the patient [11, 14, 30]. However, our analysis suggests that this design philosophy does not necessarily translate to improved joint awareness as measured by the FJS‐12. This finding could suggest that the parameters influencing a patient's perception of joint awareness are complex and may not be solely dependent on the biomechanical design of the bearing [2].

A recent meta‐analysis by Hantouly et al. using 70 RCTs found that there was no difference between MB or FB TKA at short‐term, mid‐term and long‐term follow‐ups in all outcome measures including all‐cause revision rate, aseptic loosening, oxford knee score (OKS), knee society score (KSS), Hospital for Special Surgery score (HSS score), maximum knee flexion, radiographic lucent lines and radiographic osteolysis [7]. Similar findings were reported by other meta‐analyses, such as Migliorini et al., who found no significant differences between MB and FB TKA in terms of PROMs, including OKS, WOMAC and KSS, as well as clinical outcomes like revision rates and aseptic loosening using 74 RCTs [13]. Similarly, Chen et al. reported no significant differences between MB and FB TKA in terms of functional scores, revision rates, and radiographic outcomes in long‐term follow‐up, further reinforcing that MB‐TKA does not confer a clear advantage over FB‐TKA in most clinical outcomes [4]. However, Wang et al. suggested that MB‐TKA might offer superior mid‐ to long‐term Knee Society Scores and range of motion, though no differences were found in implant survivorship or reoperation rates [27].

In this study, another PROM (FJS‐12) was evaluated, which was recently introduced to detect subtle differences in patients with good to excellent outcomes following TKA, where the available PROM tools were unable to discriminate between the two groups due to ceiling effects [12]. The literature and our study do not prove the theoretical advantages of the MB insert over its FB counterpart [5, 17, 26, 28, 31]. Given that FB‐TKA generally has lower costs and is widely used due to its simpler design, the results of this study suggest that the use of FB‐TKA might be a more reasonable choice [3].

This systematic review is subject to several limitations that must be acknowledged. First, inherent in any meta‐analysis is the potential for publication bias, although Egger's test was used to reduce publication bias. Second, the studies included in our analysis may have varied in terms of methodological quality, patient demographics and follow‐up duration, which could affect the generalizability of the results and cause the heterogeneity observed in this study. The random effects model was used to reduce the effect of heterogeneity. Additionally, while the FJS‐12 is a validated tool for assessing joint awareness, it is possible that it does not encompass all aspects of a patient's postoperative experience. Third, the alignment strategy in three studies was unknown and there are several reports that indicated alignment strategy might affect joint awareness and FJS. Finally, due to the evolving nature of TKA designs and surgical techniques, our findings may have limited applicability to future advancements in this field. Therefore, while our study provides valuable insights into the comparison of MB‐TKA and FB‐TKA, these limitations should be considered when interpreting the results and applying them to clinical practice.

## CONCLUSION

In conclusion, our findings challenge the assumption that an MB‐TKA leads to better patient‐perceived outcomes in terms of joint awareness. There was no significant difference in regard to FJS‐12 between bearing designs. The results advocate for a broader understanding of what influences joint awareness post‐TKA and suggest that future research should explore a wider range of factors that contribute to patient satisfaction and quality of life.

## AUTHOR CONTRIBUTIONS

Mohammad Poursalehian contributed to the study conception and design, analyzed data, wrote the first draft of the manuscript and edited it. Yeganeh Pakbaz contributed to the study design and data collection. Seyed Mohammad Javad Mortazavi supervised the project, validated data and revised the manuscript. All authors commented on previous versions of the manuscript and revised it. All authors read and approved the final manuscript.

## CONFLICT OF INTEREST STATEMENT

The authors declare no conflicts of interest.

## ETHICS STATEMENT

The study was systematic review and was exempt from Institutional Review Board.

## Supporting information

Supporting information.

Supporting information.

## Data Availability

The data that support the findings of this study are available as supplementary data.
