# Supplementary material for: Mobile bearing total knee arthroplasty does not lead to better joint awareness compared to fixed bearing design: A systematic review and meta‐analysis
Source: J Exp Orthop. 2024 Dec 15;11(4):e70110. doi: 10.1002/jeo2.70110 (PMC11646548; doi:10.1002/jeo2.70110)
Supplement: Supplementary file 1 — Supporting information. [file JEO2-11-e70110-s001.docx]

| Database | String | Number of Results |
| --- | --- | --- |
| Pubmed | bearing AND ("total knee" OR TKA) AND (FJS OR "Forgotten Joint Score") | 36 |
| Embase | ('bearing'/exp OR 'bearing') AND ('total knee arthroplasty'/exp OR 'TKA') AND ('forgotten joint score'/exp OR 'FJS') | 41 |
| Scopus | TITLE-ABS-KEY ( bearing AND ( "total knee" OR TKA ) AND ( **fjs** OR "Forgotten Joint Score" ) ) | 37 |
| Wos | ALL=(bearing AND ("total knee" OR TKA) AND (FJS OR "Forgotten Joint Score")) | 43 |
